# Upgrading Pyrolytic Oil via Catalytic Co-Pyrolysis of Beechwood and Polystyrene

**DOI:** 10.3390/molecules28155758

**Published:** 2023-07-30

**Authors:** Yehya Jaafar, Gian Carlos Arias Ramirez, Lokmane Abdelouahed, Antoine El Samrani, Roland El Hage, Bechara Taouk

**Affiliations:** 1 INSA Rouen Normandie, University Rouen Normandie, Normandie Université, LSPC, UR 4704, F-76000 Rouen, France; yehya.jaafar@insa-rouen.fr (Y.J.); gian.arias_ramirez@insa-rouen.fr (G.C.A.R.); lokmane.abdelouahed@insa-rouen.fr (L.A.); 2 Laboratory of Geoscience, Georesources and Environment (L2GE) Campus Fanar EDST, Faculty of Science, Lebanese University, Fanar, Jdeidet P.O. Box 90656, Lebanon; antoineelsamrani@ul.edu.lb; 3 PR2N-EDST, Laboratory of Physical Chemistry of Materials (LCPM), Campus Fanar, Faculty of Sciences II, Lebanese University, Fanar, Jdeidet P.O. Box 90656, Lebanon; roland_hage85@hotmail.com

**Keywords:** co-pyrolysis, polystyrene, beech wood, Fe/Ni-ZSM-5 catalyst

## Abstract

This study aims to investigate the catalytic co-pyrolysis of beech wood with polystyrene as a synergic and catalytic effect on liquid oil production. For this purpose, a tubular semi-continuous reactor under an inert nitrogen atmosphere was used. Several zeolite catalysts were modified via incipient wetness impregnation using iron and/or nickel. The liquid oil recovered was analyzed using GC-MS for the identification of the liquid products, and GC-FID was used for their quantification. The effects of catalyst type, beechwood-to-polystyrene ratio, and operating temperature were investigated. The results showed that the Fe/Ni-ZSM-5 catalyst had the best deoxygenation capability. The derived oil was mainly constituted of aromatics of about 92 wt.% for the 1:1 mixture of beechwood and polystyrene, with a remarkably high heating value of around 39 MJ/kg compared to 18 MJ/kg for beechwood-based bio-oil. The liquid oil experienced a great reduction in oxygen content of about 92% for the polystyrene–beechwood 50-50 mixture in comparison to beechwood alone. The catalytic and synergetic effects were more realized for high beechwood percentages as a 75-25 beechwood–polystyrene mix. Regarding the temperature variation between 450 and 600 °C, the catalyst seemed to deactivate faster at higher temperatures, thus constituting a quality reduction in the pyrolytic oil in high-temperature ranges.

## 1. Introduction

Fuels based on fossil fuels have been the main energy source over the last century, especially in the transportation sector. As the petroleum reserves are running dry, and due to severe environmental issues, a new source of fuel should be considered. The development of green renewable fuel is essential to relieve the pressure of burning fossil fuels and to reach the grand goal of carbon neutrality by 2050 [1,2]. Bio-oils from the pyrolysis of lignocellulosic biomass have received growing attention as a fuel substitute [3,4,5,6]. Unfortunately, this type of bio-oil cannot be a direct fuel substitute due to its high acidity and oxygen content of around 43 wt.% [7]. The elevated oxygen content reduces the heating value, in addition to its corrosiveness and instability [8].

Several approaches have been considered to improve the quality of bio-oil, such as catalytic deoxygenation [9,10], hydrodeoxygenation [11,12], and esterification [10]. Catalytic fast pyrolysis (CFP) of lignocellulosic biomass involves the passing of pyrolysis vapor through the pores of the catalyst structure, converting a fraction of the vapor into desirable aromatics and olefins [11,12]. This method has been conducted extensively by researchers since it reduces the oxygen content without any use of hydrogen, and it can be applied in the same reactor [13,14,15,16]. Nevertheless, this hydrogen deficiency limits the extent of deoxygenation reactions [17]. Catalytic pyrolysis of lignocellulosic biomass can also produce bio-oils of about 5 to 10 wt.% hydrocarbonaceous compounds, but the oxygen content is still high [11,18,19].

The hydrogen deficiency of most biomass can be compensated by co-feeding processes with a hydrogen-rich source such as plastic materials [17,20]. It has been shown that co-feeding biomass with synthetic polymers in catalytic pyrolysis could positively influence the performance of pyrolysis and the quality of products formed [21,22,23,24]. This addition could mimic hydrodeoxygenation by supplying the needed hydrogen at atmospheric pressure with minor modifications and an attractive performance/cost ratio. Furthermore, this method could consume plastics and limit their disposal, especially those as challenging to recycle as polystyrene [25]. For instance, polystyrene can be converted to styrene via pyrolysis with very high yields (>60 wt.%) [26,27].

Several researchers supported the performance of zeolites, especially ZSM-5 in deoxygenation potential and aromatic selectivity due to its acidity and pore dimension [17,21,22]. It has been proven that modifying zeolite with metal impregnation has many advantages [28,29,30]. W. Yao et al. [31] investigated the catalytic co-pyrolysis of pine wood with low-density polyethylene in a semi-continuous pyroprobe. The catalyst was mixed with the feed with a catalyst-to-feed ratio of 15 at 550 °C. In this study, the authors used a ZSM-5 catalyst modified with a mixture of nickel (Ni) and phosphorus (P) 3%/2% P/Ni-ZSM-5 and with phosphorous alone 2% P-ZSM-5. The results showed an increase in olefins and aromatics from 43 wt.% for conventional ZSM-5 to about 53–54 wt.% for P- and P/Ni-ZSM-5. The word aromatics in this domain usually stands for only hydrocarbon aromatic compounds in this paper, excluding phenols and other oxygenated aromatic compounds. Lin et al. [32] utilized a similar modification of ZSM-5. The study investigated the performance of P-ZSM5 through the catalytic co-pyrolysis of poplar wood and high-density polyethylene in a quartz pyroprobe, with P-loadings varying from 0 to 10 wt.%. The effect of several parameters was studied as heating rates, temperature, residence time, and catalyst-to-feedstock ratio. It was found that the parent ZSM-5 favored aromatic production, whereas the modified P-ZSM-5 favored the formation of light aliphatic hydrocarbons. Another approach was carried out by J. Li et al. [33], where the catalytic co-pyrolysis of pine wood and low-density polyethylene using a gallium-modified zeolite in a semi-continuous microreactor at 550 °C was studied. The results showed an increase in monoaromatics of 5% in the case of Ga-ZSM-5 compared to the parent ZSM-5 by about 5 wt.%.

Previous studies discussed the effect of the modification of zeolites on the catalytic co-pyrolysis of biomass and polyolefins in particular [31,32,33]. However, the CFP of polystyrene was not extensively studied under metal-modified zeolites. Several researchers investigated the latter only over H-ZSM5, which recorded a further improvement in the quality of the bio-oil towards valuable aromatic products [34,35,36]. To propose a new catalyst modification in catalytic co-pyrolysis, this study used both iron and nickel incorporated with H-ZSM-5. Iron-based zeolites prove to be active in the catalytic pyrolysis of biomass. It has been stated that 1.4 wt.% Fe produced the largest decrease in oxygen content, from lignocellulosic to about 14 wt.% [37]. Furthermore, nickel-incorporated ZSM-5 was considered in the catalytic pyrolysis of biomass to increase the aromatic yield while increasing the hydrothermal stability of the catalyst [38]. The combination between Ni and the acids sites provides a reactive environment for improving aromatic formation [39]. This study aims to present a detailed analysis of the catalytic co-pyrolysis of polystyrene (PS) and beechwood BW (*Fagus sylvatica*) as lignocellulosic forestry residue, under iron- and nickel-modified zeolites (Fe/Ni-ZSM-5). Indeed, the pyrolysis of PS yields high-value oil, but the aim is to improve the oil obtained from lignocellulose biomass via catalyst co-pyrolysis. BW is used since it is, by far, the most used hardwood in Europe [40], hence producing a lot of beech sawdust. For that, BW must be included in any attempt to study the potential of bio-oil as an alternative fuel. The influence of different parameters, such as metal loading of the catalyst, biomass/plastic ratio, and pyrolysis temperature, on the quality and quantity of co-pyrolysis oil was investigated. At first, each feedstock (BW and PS) was used independently and as a mixture to produce a database reference of pyrolytic oils under the several synthesized catalysts. The best catalyst was chosen; then, other parameters, such as temperature and biomass-to-plastics ratio, were investigated to improve the best pyrolytic oils.

### 1.1. Catalyst Characterization

The chemical and textural properties of the synthesized catalyst are summarized in Table 1. It is noticed that there are no major variations in the SiO_2_/Al_2_O_3_ ratios due to the mild modifications and low metal loading. The actual Fe and Ni loading were slightly close to the planned percentages.

**Figure 1:**
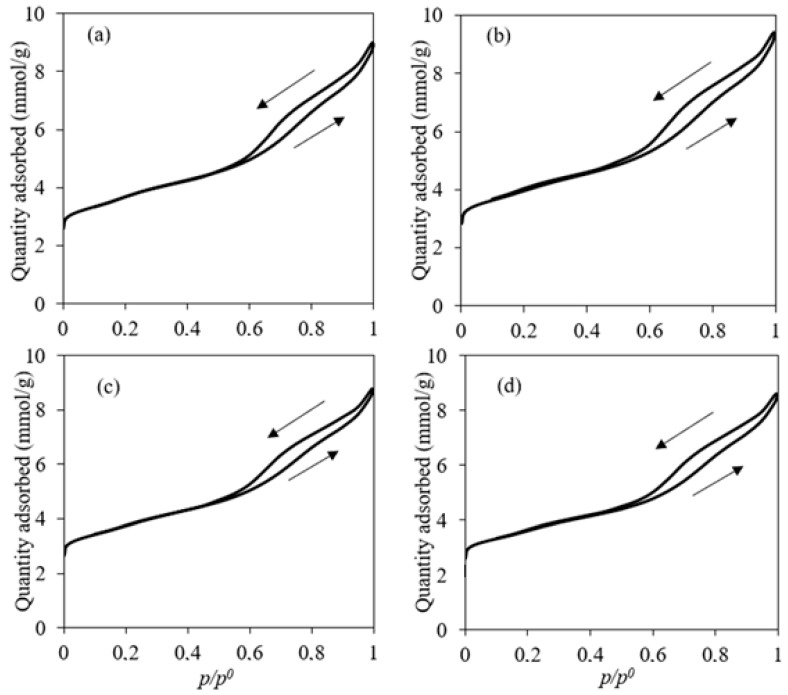
N_2_ adsorption/desorption isotherms. (**a**): ZSM-5; (**b**): Fe-ZSM-5; (**c**): Fe/Ni-ZSM-5; (**d**): Ni-ZSM-5.

**Figure 2:**
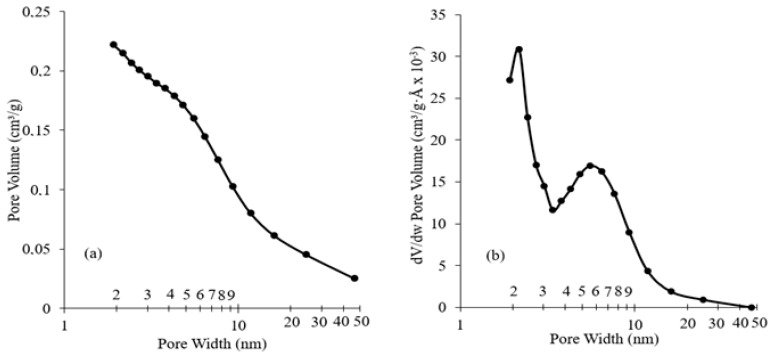
Barrett–Joyner–Halenda (BJH) pore size distributions of Fe/Ni-ZSM-5. (**a**): Cumulative pore volume, (**b**): differential pore volume.

The BET surface area attained an average value between 274 and 299 m^2^/g, with no significant variation after impregnation. The elevated surface area of all catalysts could have a good impact on the performance of the catalyst. In most cases, the higher the surface area, the better the activity. It has been shown that after impregnation, the surface area would be reduced [31]. It was not the case in this study due to the low metal loading, thus keeping the surface activity of the zeolites high. This observation was in accordance with the work of Li et al. [41], where no significant change in BET surface area was observed for an Fe/ZSM-5 content of around 3 wt.%. N_2_ adsorption–desorption isotherms of the used catalysts are illustrated in Figure 1**.** The adsorption–desorption isotherms observed for the catalysts exhibited a hysteresis loop corresponding to a type IV isotherm [42]. However, the isotherms showed a broad loop at p/p^0^ = 0.5–1.0, which can indicate the co-existence of both micropores and mesopores. This hypothesis is further verified considering Figure 2a, which describes the distribution of the cumulative pore volume relative to pore size. All catalysts showed similar behavior, so only the Fe/Ni-ZSM-5 figure is illustrated. There was no variation in the specific pore volume of the modified ZSM-5 relative to the parent after impregnation. The pore volume was approximately 0.23 m^2^/g. This could suggest that Fe and Ni were deposited mainly on the edges and external surface area of ZSM-5 [43]. The derivative of the pore volume relative to pore width was further investigated in Figure 2b. The curve showed two maximums referring to around 2 nm and 6 nm, further verifying the previous deduction of both micro- and mesopores of the prepared ZSM-5.

**Figure 3:**
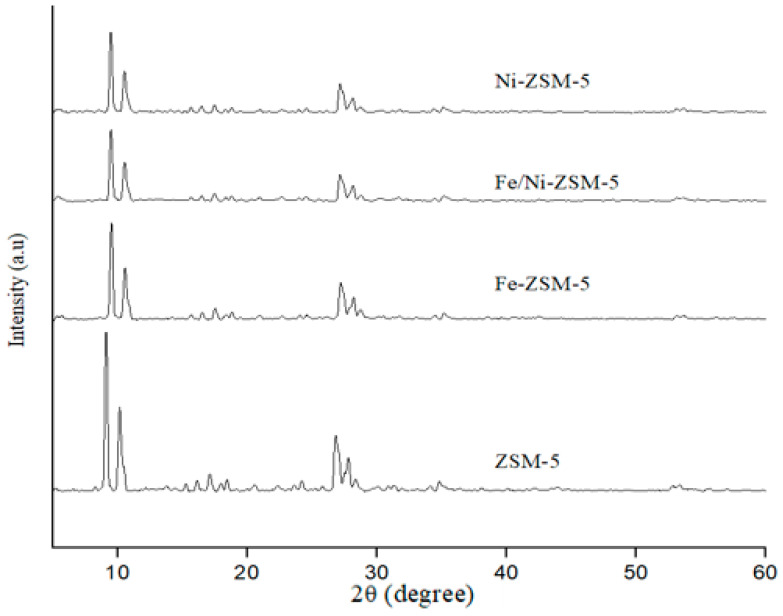
XRD patterns of the parent, Fe-, Fe/Ni-, Ni-modified ZSM-5 zeolites.

The size population distribution followed a monomodal distribution, with a mean size diameter of about 1.3 mm ± 0.4 via granulometric analysis. As for the XRD analysis, the patterns of the parent ZSM-5 showed a typical MFI zeolite structure, as shown in Figure 3. The results showed that the modification with Fe and Ni did not affect the framework structure of ZSM-5. All samples exhibited peaks at 2θ of 9° and 27°, with no additional peaks after modification. Furthermore, the intensity of the diffraction peaks was reduced slightly after impregnation, implying a slight reduction in crystallinity. This could be because during impregnation, some chemical changes may occur in the pores of the catalyst, especially after chemical impregnation followed by calcination at 550 °C [44].

**Figure 4:**
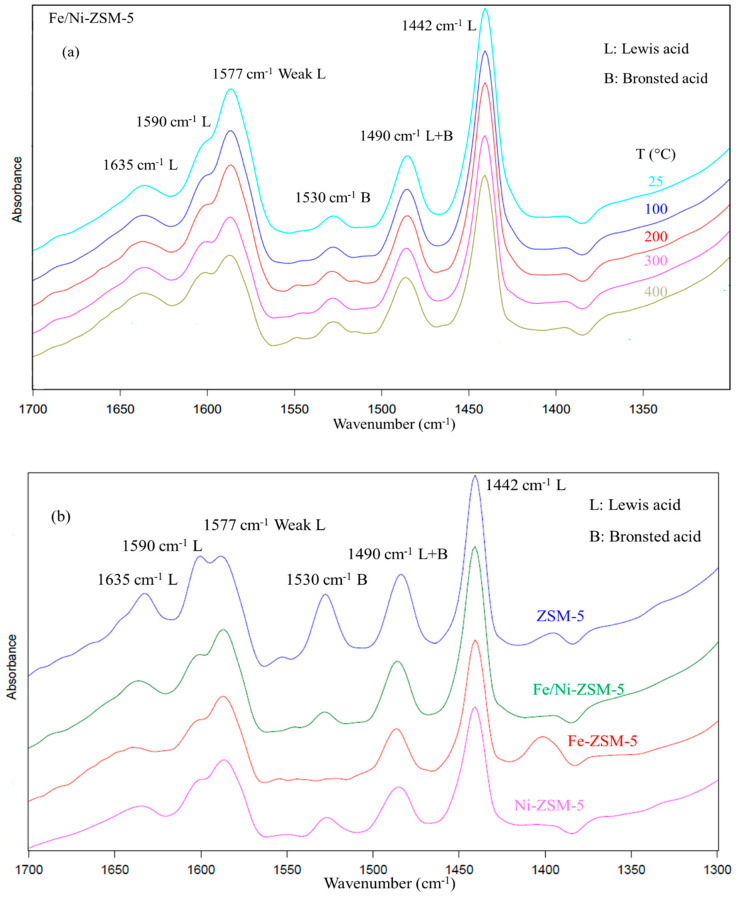
FT-IR spectra of the catalysts. (**a**) Fe/Ni-ZSM-5 at different temperatures, (**b**) comparison between the different catalysts at 300 °C.

Figure 4a represents the FT-IR spectra of adsorbed pyridine on Fe/Ni-ZSM-5 after evacuation at different temperatures. The behavior of the other catalysts as per temperature was similar. Firstly, zeolites are known to have two types of acid sites: Lewis acid and Bronsted acid sites. IR peaks at wavelengths of 1445 cm^−1^, 1590 cm^−1^, and 1620 cm^−1^ are attributed to pyridine adsorbed on Lewis acid sites, along with a weak Lewis acid site at 1577 cm^−1^, which is usually present for zeolites [45,46]. The Lewis acid sites usually come from the extra-framework of Al and O atoms [45,47]. On the other hand, Bronsted acid sites originate from the hydroxyl groups, linking Al and Si atoms, and they are identified by the IR wavebands of 1530 cm^−1^ and 1645 cm^−1^. Most of the latter wavelengths were recorded for the used zeolites with an additional peak of 1490 cm^−1^, which was ascribed to physisorbed pyridine, which can be promoted by both Lewis and Bronsted acid sites [45,47]. The intensity of the peaks formed was reduced due to the increase in temperature to 400 °C, yet pyridine was not completely removed. This behavior confirms the high strength of pyridine adsorption on the acid sites of the catalyst and highlights incomplete desorption [48].

Unlike the other characterization technique, the variation in acidity after impregnation was more pronounced. The parent zeolite was shown to have the greatest acid bands, as shown in Figure 4b. The effect of metal modification reduced the overall acidity of the catalyst, which can imply that the acid sites were partially displaced by the metal ions. This was mainly pronounced for Fe-ZSM-5, where the modification with Fe completely removed the Bronsted acid site of 1530 cm^−1^.

### 1.2. Catalytic Pyrolysis of Individual Biomass and Plastic

First, the GC-MS analysis of various pyrolytic oils was carried out to identify the present compounds in each pyrolytic oil obtained at 500 °C. The analysis was conducted for BW and PS alone and as a mixture without and with every synthesized catalyst. For instance, around 120 and 100 compounds were identified for PS and BW, respectively. The compounds identified for PS with and without a catalyst were similar, and all the compounds were aromatic hydrocarbons. However, for BW, the majority of the compounds were oxygenated compounds, yet after catalysis, new aromatic hydrocarbons were further identified as benzene, toluene, xylene, styrene, and indene.

The identified compounds were grouped into the most relevant organic families divided between oxygenated and hydrocarbon compounds to better visualize further analysis [27,49]. The oxygen content is calculated from the percentage of oxygen atoms in each oxygenated molecule. This method is detailed in a previous work [49]. The families and major products for calibration are illustrated in Table 2. A list of the major compounds is present in Table S1.

**Figure 5:**
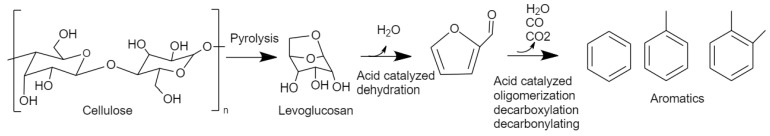
Reaction pathway of catalytic pyrolysis of biomass over ZSM-5 at 400–600 °C, adapted from R. Carlson et al. (2011) [14].

Beechwood pyrolysis produces an oil rich in acids, ketones, and carbohydrates (levoglucosan). The distribution can be observed. Carboxylic acids constituted around 35 wt.%, followed by carbohydrates and ketones with respective concentrations of 17 wt.% and 15 wt.%. It was considered that BW was chemically composed of 42 wt.% cellulose, 37 wt.% hemicelluloses, and 19 wt.% lignin [50]. This would explain the domination of carbohydrates and acids on the liquid bio-oil since they are the dominant species in the independent pyrolysis of cellulose and hemicellulose, respectively, whereas phenols and guaiacols are derived from lignin [51]. As can be seen, BW pyrolysis has no hydrocarbon aromatic production, thus leaving the oil with high oxygen content and low LHV of around 41 wt.% and 18 MJ/kg, respectively, as shown in Table 3. Table 3 shows the variation in the properties of the bio-oil after catalysis. In contrast to BW pyrolysis, catalytic pyrolysis showed the formation of aromatic compounds. The total aromatics were around 7 wt.% for the catalytic pyrolysis under the parent ZSM-5. This production of aromatics mainly came at the expense of levoglucosan (derived from cellulose) without any major variation in the other compounds. This can be explained by the transformation of the latter compound into aromatic compounds via catalytic decarbonylation, decarboxylation, dehydration, oligomerization, and isomerization on the surface of the catalysts, as explained in Figure 5 [14]. On the other hand, phenolic compounds and guaiacols seemed to increase after catalysis. The reason for that lies in the aromatic selectivity and pore structure of ZSM-5. Phenols and guaiacols have a similar structure to monoaromatic compounds, so an increase in those compounds can be due to the transformation of aldehydes, ketones, carbohydrates, and furans into oxygenated aromatics in the pores of the catalyst without the deoxygenation step. The LHV and oxygen content varied slightly to be around 20 MJ/kg and 36 wt.%, respectively. This variation can be due to catalytic oxygen elimination mainly into CO and CO_2,_ represented by an increase in gas yield that is mainly CO, CH_4_, and CO_2_.

The effect of the different metal loadings on the performance of catalytic pyrolysis of BW was investigated, and the results are shown in Table 3. The parent ZSM-5 showed the best selectivity towards aromatics and PAH compared to other modifications. This suggests that the modification by metals on the original zeolite inhibits aromatic formation [37], yet the inhibition is minimal due to low metal loading. The char yield was not affected since it was produced before catalysis, and coking was somehow similar.

As for the individual catalytic pyrolysis of polystyrene (Table 4), the effect was like a typical cracking reaction. At first, the pyrolytic oil was mainly composed of monoaromatic hydrocarbons of about 77 wt.%, mainly the styrene monomer. The heating value was elevated (about 40 MJ/kg) with no oxygen due to the hydro-carbonaceous nature of polystyrene. The liquid yield was almost total, with no gases or char, which was confirmed in a previous study [27]. On the other hand, the catalytic effect on polystyrene liquid oil enhanced the cracking and dissociation reaction; thus, the lighter hydrocarbon was higher for all four catalysts, with an advantage to the parent zeolite. This reinforces the former deduction that the selectivity of the parent zeolites towards aromatics was reduced through impregnation. However, PAH was transformed into lighter aromatics, yet not gases. The gas yield was still minimal. This would further support the high selectivity of zeolites toward aromatics [52]. Ultimately the reduction in liquid yield was at the expense of coke.

### 1.3. Catalytic Co-Pyrolysis of Biomass and Plastic

**Figure 6:**
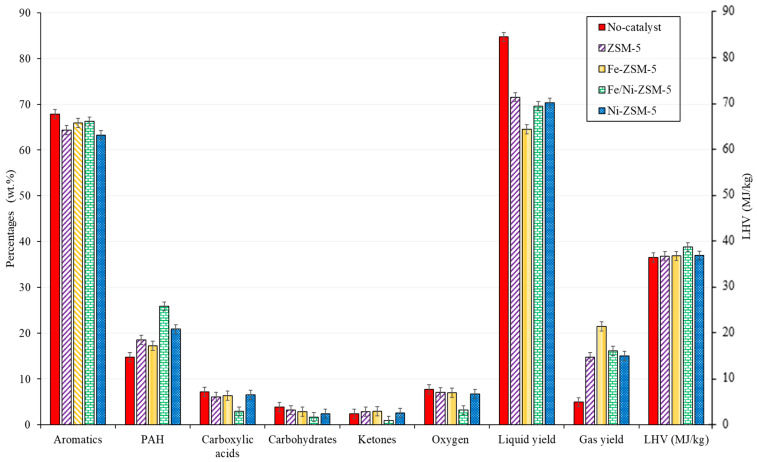
Comparison of the used catalyst relative to the major families and main properties of BW-PS (50-50) liquid oil (wt.%) 500 °C.

The catalytic co-pyrolysis of biomass and plastic was investigated, as shown in Figure 6. The effect of metal loading and the distribution of main properties were also studied. At first, the non-catalytic co-pyrolysis of biomass and plastics yielded an oil rich in aromatics with a low oxygen content and high heating value of around 8 wt.% and 37 MJ/kg, respectively. However, the oxygen content should be further reduced to have the potential to be used as fuel directly. On the other hand, despite the oxygen content, the total acids and oxygenated compounds could cause several problems, such as corrosion and chemical instability. For instance, biomass bio-oil exhibits a pH value of around 2–3; the value differs between each type of biomass but, in general, the oil is highly acidic [8]. Although the concentration of acids was reduced significantly, the pH would not reach neutral levels.

**Figure 7:**
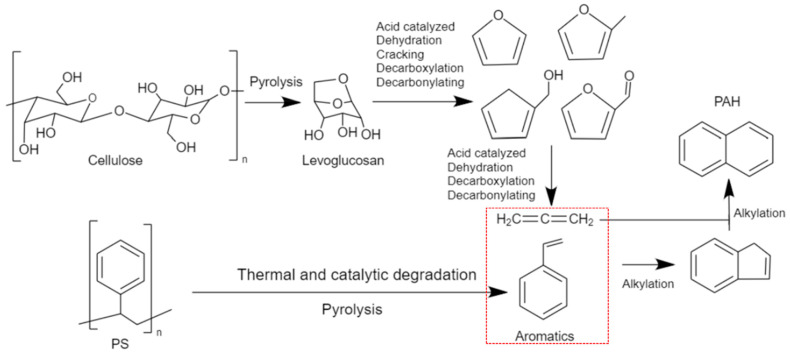
Reaction pathway of catalytic co-pyrolysis of polystyrene and biomass over ZSM-5, adapted from Cheng and Huber (2012) and Dorado et al. (2015) [53,54]. The red box highlights intermediate products.

After catalysis, for all the prepared catalysts, the liquid yield was significantly reduced from 85 wt.% to 70 wt.% at the expense of coking and gas production, which is typical for any catalytic cracking. On the other hand, the polycyclic aromatic hydrocarbons (PAHs) increased after catalysis. This increase was followed, in general, by a decrease in single aromatic and other oxygenated compounds. This increase in PAH can be supported by the mechanism shown in Figure 7. The reaction pathway was proposed between PS and biomass components (cellulose) in the presence of a ZSM-5 catalyst in a temperature range of 300–650 °C [53,54]. Naphthalene formation and other PAH could be obtained through Diels–Alder reactions of monoaromatics and furans (originated from levoglucosan) through a series of alkylation reactions [53]. The mechanism suggests the reaction pathway of cellulose with PS, which is considered one of the main constituents of BW.

Regarding the metal loading of the catalyst, all the metal loading yielded approximately the same outcome, except for the Fe/Ni-ZSM-5, regarding deoxygenation. For instance, the latter catalyst exhibited a slight reduction in oxygen, with no major change within the compositional analysis of the pyrolytic oil. The co-impregnation of iron and nickel onto the surface of the catalyst had a great effect on the oxygen content of the pyrolytic oil during co-pyrolysis. The oxygen content reached very low values of around 3 wt.%, with a decrease in acids and other oxygenated compounds. The reduction in oxygen content was close to that of Dyer et al. [55], who studied the catalytic co-pyrolysis of waste wood with polystyrene under ZSM-5. The oxygenated compounds reached 2% relative to the total ion chromatography peak. The value is low, but it is not accurate for comparison since there was no calibration to obtain the real value. The effect of the Fe/Ni-ZSM-5 catalyst was seen in co-pyrolysis but not in the pyrolysis of BW, which implies that the catalyst further reinforces the synergy between PS and BW. The hydrogen supply from the plastics was best utilized for this catalyst. This could be due to the interaction between both metals (Fe and Ni) and the higher metal loading compared to the other catalysts.

### 1.4. Catalytic Co-Pyrolysis under Fe/Ni-ZSM-5

**Figure 8:**
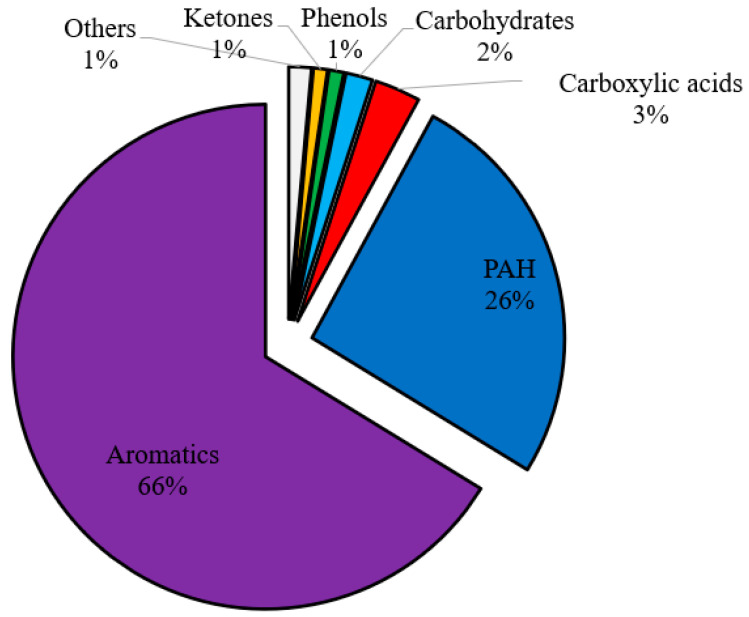
Chemical families of BW-PS 50-50 (Fe/Ni-ZSM-5) liquid oil at 500 °C.

To delve deeper into the quality of the bio-oil produced, several parameter tests were conducted on the pyrolytic oil derived from the Fe/Ni-ZSM-5 catalyst. The oil was mainly composed of aromatics of around 92 wt.%, of which 66 wt.% are monoaromatics and 26 wt.% are PAH (Figure 8). The oil exhibited very low acid and other oxygenated compound content. For this reason, this type of oil could be used as a possible substitute for petroleum fuel due to its high heating value and low oxygen and acids content. However, several parameters should be investigated before recommending the oil for automotive fuels (gasoline and diesel), such as octane and cetane number, viscosity, density, distillation profile, etc. [56]. These parameters could not be calculated in this study since a solvent was used to collect the oil; the derived oil should be pure to be able to calculate the latter properties. Yet, regarding the octane number, the oil should have a high octane number due to the dominance of aromatics [27]. Nevertheless, PAH (26 wt.%) should be removed so that the oil could be used as gasoline or as a gasoline blend; otherwise, it could be burned directly as heating fuel.

**Figure 9:**
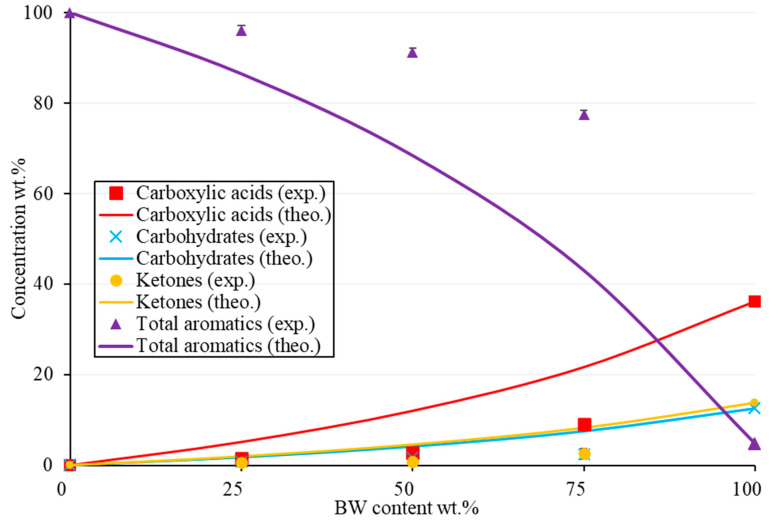
Distribution of the major families of BW-PS (Fe/Ni-ZSM-5) liquid oil as experimental and theoretical concentrations relative to BW content at 500 °C (wt.%).

The effect of the BW/PS ratio on the family’s distribution is shown in Figure 9. The synergetic effect was also discussed by representing the experimental and theoretical values of each family computed via Equation (3). The theoretical values vary relative to each BW percentage in the feed. The synergy gave a positive effect by increasing aromatics and reducing the oxygenated compounds. For instance, for the total aromatic concentrations, the effect of adding plastics improved the yield by around 24 wt.% for the 50-50 mix. This effect was better observed for higher BW content. The synergy showed an increasing trend as BW content increased. For example, the difference between the theoretical and experimental concentrations of total aromatics was around 10 wt.% for the 25-75 BW/PS mix compared with 34 wt.% for the 75-25 BW/PS mix. Therefore, this was good since the objective of catalytic co-pyrolysis is to enhance the properties of bio-oil from biomass pyrolysis, so the more one uses a natural, renewable, reliable, and carbon-neutral source, the greater the advantage towards the environment.

**Figure 10:**
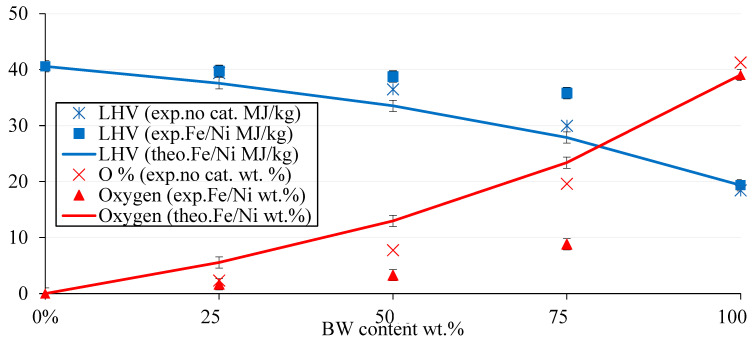
The evolution of LHV (MJ/kg) and O% (wt.%) in BW-PS (Fe/Ni-ZSM-5) liquid oil as per BW content at 500 °C.

The synergetic and catalytic effects on the LHV and oxygen content are further discussed in Figure 10 and Table 5. The synergetic effect was also evident regarding the calorific value of liquid oil. The latter conclusion was further reinforced; the synergy was more significantly realized at high BW content. On the other hand, compared to co-pyrolysis without a catalyst, the trend showed that the LHV and oxygen content of the liquid oil for the 50-50 mix was like that of the 75-25 mix with a catalyst, and the 25-75 mix without a catalyst was like that of the 50-50 mix with a catalyst. For instance, the LHV and oxygen content were around 37 MJ/Kg and 8 wt.%, respectively, for the 50-50 mix without a catalyst, which was similar to 36 MJ/Kg and 9 wt.% for the 75-25 mix with a catalyst. Hence, the addition of a catalyst mimicked the effect of increasing hydrocarbon feed to the mixture, without any significant change in the properties of the liquid oil. Referring to Table 5, the percentage increase in LHV from BW virgin bio-oil attained its maximum for the catalytic pyrolysis with Fe/Ni-ZSM-5 for the 50-50 mix. The latter increase was about 111%, accompanied by deoxygenation of around 92%.

The derived liquid oils were then compared to conventional petroleum fuels to study the possible end-use of each product. The most promising liquid fuel was from the catalytic co-pyrolysis of two different feedstock proportions, 50-50 and 75-50. The first showed a further decrease in oxygen content, making the oil more suitable to be used as automotive fuel. However, due to the high concentrations of aromatics, the oil cannot be used as diesel oil so it should be fractionated to match the gasoline carbon range; then, it could be used with some minor modifications [57]. The latter pathway equally favors PS and BW consumption as feedstock, yet to fully benefit from the word’s forestry residue, the second option is still feasible. Furthermore, the bigger the use of a renewable carbon-neutral energy source such as BW, the more sustainable and environmental the process becomes. The oxygen content is higher compared to that of the 50-50 mix; still, it could be used in furnaces for burning or to be blended with gasoline with adequate proportions.

Ultimately, a temperature analysis was carried out to specify the optimum operating temperature. The main composition and properties of the 50-50 catalytic co-pyrolysis oil relative to temperature are summarized in Table 6. Regarding the product yield, the liquid and char yield decreased as per temperature, accompanied by an increase in the gaseous yield. This behavior is typical for cracking reactions. Nevertheless, coking on the catalyst surface increased with temperature, and the oxygen content also increased, especially from temperatures of 550 °C and above. This suggests that the catalyst deactivated and lost its deoxygenation potential at higher temperatures (≥550 °C). This behavior could be attributed to the catalyst’s performance. The catalyst was calcined at a temperature of 550 °C; therefore, it is expected to have a decrease in performance at higher temperatures. The same behavior was reported by Zhang et al. [17] for the co-pyrolysis of pine sawdust and polyethylene. They reported lower catalytic performance when co-pyrolysis was conducted at a temperature of 650 °C, above the calcination temperature of 600 °C. Ultimately, the optimum operating temperature for the catalytic co-pyrolysis would be 500 °C, where the liquid oil had the best quality, and the liquid yield was relatively high.

## 2. Experimental Section

### 2.1. Materials

The virgin polystyrene (PS) was supplied by Goodfellow company (Huntingdon, UK), and beechwood was supplied by ETS Lignex Company (Patornay, France). PS was milled and sieved with a 2 mm average mesh, whereas the average particle size of beechwood was about 0.4 mm, as supplied. The conventional ZSM-5 in its proton form (SiO_2_/Al_2_O_3_ ratio of 38, specific surface area of about 250 m^2^/g, and 5 nm of pore size) was acquired from ACS materials (Pasadena, CA, USA) as 3 mm diameter pellets. The catalyst was milled and sieved to 0.6 mm and 1.18 mm particle size. The upper range ensures the proper stacking of adequate quantities of catalyst in the reactor, whereas the lower range ensures gas flow without the risk of clogging and pressure buildup. Iron nitrate salt, ferric (III) nonahydrate (Fe(NO_3_)_3_·9H_2_O), nickel nitrate salt, and nickel (II) nitrate hexahydrate (Ni(NO_3_)_2_·6H_2_O) were purchased from Panreac AppliChem (Darmstadt, Germany) and Alfa Aesar (Kandel, Germany), respectively.

The elemental analysis was obtained under oxidizing atmosphere by the combustion of BW and PS samples in the presence of tungstic anhydride at a high temperature for about 20 s using a CHN elemental analyzer Flash 2000 (Thermofisher Scientific, Waltham, MA, USA). The calculation of the percentage of each element was analyzed using the “Eager 300” software (version 2.2) (Table 7).

Thermogravimetric (TGA) measurements were achieved using an SDT/Q600-TA analyzer for the ultimate analysis (Table 7). The heating rate was 5 °C/min under a nitrogen flow rate of 50 mL/min and at atmospheric pressure.

### 2.2. Catalyst Preparation

**Figure 11:**
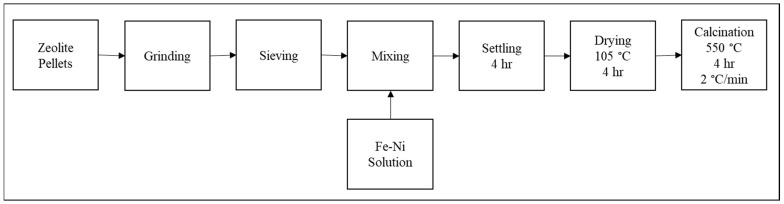
Catalyst preparation procedure.

A set of Fe/Ni-modified ZSM-5 was prepared by impregnating around 50 g of ZSM-5. Iron and nickel nitrate salts were used to prepare three metal loading: 1.4 wt.% Fe/ZSM-5, 1.4 wt.% Ni/ZSM-5, and (1.4 wt.% Fe and 1.4 wt.% Ni)/ZSM-5. Then, the weighed nitrates were dissolved in a 40 mL deionized water solution. Impregnation with 50 g of ZSM-5 was carried out using incipient wetness impregnation method. Impregnation was performed at ambient temperature for about 4 h. The 3 impregnated samples were then dried overnight at 105 °C. Finally, the obtained catalysts along with the parent ZSM-5 were calcinated at 550 °C for 4 h, with a heating rate of 2 °C.min^−1^ in an oxidative atmosphere (air). The catalysts were then noted as ZSM-5, 1.4% Ni-ZSM-5, 1.4% Fe-ZSM-5, and 1.4/1.4% Fe/Ni-ZSM-5. For better understanding, a detailed schema concerning the catalyst preparation steps is described in Figure 11.

### 2.3. Catalyst Characterization

The actual Fe and Ni contents were computed using inductively coupled plasma optical emission spectroscopy (ICP-OES) from Thermofisher Scientific (Waltham, MA, USA) and further verified via X-ray fluorescence (XRF) technique using Thermo-fisher scientific Niton™ XL2. X-ray diffraction (XRD) patterns were recorded by a D5000 diffractometer at 40 kV and 40 mA with CuKα radiation in a range of 2θ from 5 to 100 degrees. Particle size distribution was analyzed using Horiba Partica laser scattering LA-950V2 (Kyoto, Japan).

The textural properties’ BET surface area and pore size of the catalysts were characterized by N_2_ adsorption–desorption at −196 °C on a Micromeritics Gemini VII (Georgia, USA). Before analysis, the samples were degassed overnight at 200 °C. Nitrogen adsorption–desorption isotherms were obtained over a wide range of relative pressure (P/P_0_) from 0.01 to 0.99.

The acidity of the catalyst was measured using infrared spectroscopy of adsorbed pyridine. Pyridine was chosen as a probe molecule to examine the acidity of zeolites [45].

The FT-IR spectra (Fourier-transform infrared spectroscopy) of each pyridine adsorbed catalyst as well as of pure ZSM-5 were recorded using diffuse reflectance infrared Fourier-transform (DRIFT) technique on an FT-IR spectrometer by Perkin Elmer in the 4000–400 cm^−1^ wavenumber range. The samples were prepared in powder form and diluted with KBr (10% dilution) to obtain a smooth and clear spectrum.

For the acidity test procedure, each sample was degassed at 400 °C for 4 h using a flow of 50 mL/min of nitrogen for evacuation. Pyridine adsorption was followed at room temperature for 60 min to assure sample saturation. Desorption of pyridine was performed by heating the sample to the desired temperature, 10 min of evacuation at the set temperature, and cooling the sample at 25 °C before recording the spectrum. This procedure was repeated for 100, 200, 300, and 400 °C.

### 2.4. Experimental Methods

#### 2.4.1. Pyrolysis Experimental Setup

**Figure 12:**
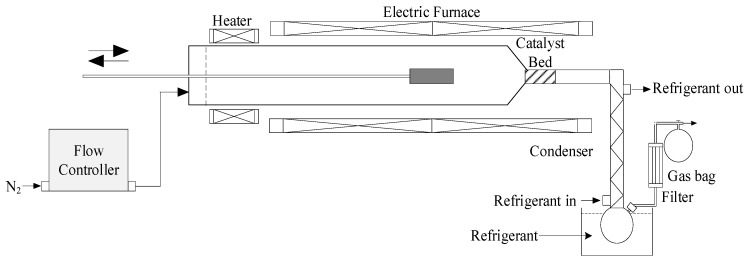
Schematic setup of the tubular reactor.

The pyrolysis reactions were performed in a semi-continuous tubular reactor with a quartz tube (ϕ = 50 mm, L = 1050 mm) inserted horizontally (Figure 12) (Pyrolysis zone). The catalyst was placed downstream in the narrower part of the reactor. The catalyst bed was 1 cm in length and 2.5 cm in diameter, weighing around 3.7 g (5 cm^3^ volume catalysis zone). Nitrogen was used as a carrier gas with a flow rate of about 400 mL/min. To insert the samples, a stainless-steel sample carrier “spoon” was placed and pushed at the inlet of the reactor, while the other side was connected to a flask and a condenser for liquid collection at a condensation temperature of around 5 °C. The non-condensable gases were also collected using a Tedlar gas bag. At first, the catalyst and BW were dried in an oven at 105 °C for 2 h to eliminate excessive moisture. Then, a typical 3 g sample of plastic and/or biomass was loaded into the sample carrier (1.2:1 catalyst-to-feed ratio). After the required temperature was attained, the sample carrier was pushed inside the heating zone to achieve isothermal conditions. Liquid oil was recovered from the condenser and the flask using acetone as solvent. The total mass balance was not achieved since there was some accumulation of bio-oil inside a part of the experimental set-up for catalytic co-pyrolysis, and it was, therefore, very difficult to fully collect the liquid products. The gas yield was measured using GC-FID/TCD, char was weighed, and coke was calculated by the difference in catalyst mass. However, because of the difficulty in determining the amount of oil accurately, its yield was considered as (100%—all other yields). For a typical trial, the experiment was repeated 3 times with a maximum error not exceeding 1%, both for temperature and experimental results. All the experimental conditions utilizing a catalyst were also carried out without a catalyst for comparison purposes. Ultimately, one catalyst (Fe/Ni-ZSM-5) was chosen to carry out feedstock ratio and temperature analysis. This catalyst was chosen since it gave the best results during catalytic co-pyrolysis. The performed experiments are summarized in Table 8.

#### 2.4.2. GC-MS Analysis for Liquid Products

Gas chromatography–mass spectrometer instrument GC-MS (Perkin Elmer Mass spectrometer Clarus^®^ SQ 85), with an Agilent VF-1701 ms column (60 m × 0.25 mm I.D × 0.25 µm film thickness) was used to identify the compounds in the pyrolytic oil. A flow of 1 mL/min of helium was used as a carrier gas along with a detector temperature of 250 °C and an MS electron ionization energy of 70 eV. The detection was set to full-scan mode. About 1 µL of the sample was injected at 250 °C (split ratio 30:1). The oven temperature was then held at 45 °C for 4 min and heated to 240 °C, with a heating rate of 4 °C/min for 20 min. Ultimately, Varian WS (WorkStation) and NIST 2.3 software were used to identify the MS spectrum for each compound detected. Kovats retention indices of the identified components were computed and confirmed to reference values to validate their identity.

#### 2.4.3. GC-FID Analysis for Liquid Products

GC-FID Scion 456-GC Bruker apparatus was used to quantify the components. The column, temperature program, and method were identical to those of the GC-MS. The identified chemical compounds were then classified and grouped into chemical families based on their functional groups. For calibration, one pure reference compound was selected for each family. Several standard solutions of the reference compounds of different concentrations were prepared and dissolved in acetone. Four-point calibration curves (with R^2^ > 0.99) were established for these pure compounds. For each chemical group, calibration data of the reference compound were used to derive the concentration of all compounds inside the same family by computing the relative response factor between each compound and the reference compound in the same family. This method takes into consideration the structural and molecular differences between compounds to derive a difference in the response factor. The effective carbon number method (ECN) was used to correct the relative response factor [58].

#### 2.4.4. GC-FID/TCD Analysis of Non-Condensable Gases

The non-condensable gases were analyzed via a Perkin Elmer^®^ Clarus 580 gas chromatograph equipped with a methanizer, hydrogen generator, Shincarbon St 100 120 column (1 m × 1 mm ID × 1/16 in OD), flame-ionization detector (FID), and thermal conductivity detector (TCD). The oven temperature program was set in a range of 100–180 °C. The FID detects hydrocarbons, whereas the TCD for allowed measuring H_2_ and N_2_. The methanizer was used to convert CO and CO_2_ to methane for detection.

#### 2.4.5. Lower Heating Value (LHV) and Synergism between Biomass and Plastic

Dulong equation [59] was used to calculate the LHV and HHV of each pyrolytic oil:

*HHV_fuel_* = 33.80 *x_C_* + 144.20 *x_H_* − 18.03 *x_O_*   (MJ/kg) (1)

*LHV_fuel_* = *HHV_fuel_* − 21.96 *x_H_*   (MJ/kg) (2)

*x_C_*, *x_H_*, and *x_O_* are the respective weight percentage of carbon, hydrogen, and oxygen.

The synergetic effect was investigated to better understand the interactions between biomass and plastic with and without catalysts. Equation (3) was used to calculate the theoretical properties and concentration (*x_theo_*) based on the weighted average sum of each feedstock and their corresponding liquid or gas yield. The equation was developed further to compute the expected masses.

(3) $$x_{theo}=\frac{m_{theo}}{m_{total}}=\frac{x_{p}y_{p}w_{p}+x_{b}y_{b}w_{b}}{y_{p}w_{p}+y_{b}w_{p}}$$

where *m_the_*_o_ is the theoretical mass produced and *m_total_* is the total theoretical mass of the liquid oil. *x_p_* and *x_b_* are the desired product mass percentage, *y_p_* and *y_b_* are the pyrolytic liquid or gas yield, and *w_p_* and *w_b_* are the weight percentage of the biomass and plastic in the biomass–plastic mix, respectively. The significance of this equation is that we could compare the values directly to the experimental value. If *x_exp_* > *x_theo,_* the synergy is positive for that product, *x_exp_* < *x_theo_* negative synergies, and, finally, *x_exp_* ≈ *x_theo_* no synergy.

## 3. Conclusions

The catalytic co-pyrolysis of beechwood and polystyrene creates a good opportunity for the valorization of a widely known forestry product and the reduction in an environmentally hazardous plastic such as polystyrene. After deeper analysis, the derived oil mainly constituted about 92 wt.% of aromatics for the 1:1 mixture of beechwood and polystyrene with a high heating value of around 39 MJ/kg compared to 18 MJ/kg for beechwood bio-oil. The liquid oil experienced a great reduction in the oxygen content from 41 wt.% (beechwood) to just 3 wt.% for the polystyrene–beechwood 50-50 mixture, which is a great reduction of about 92%. The catalytic and synergetic effects were more effective with high biomass proportions. This could increase the utilization of a renewable and reliable carbon-neutral energy source in an economically feasible process. The derived oil could be used as alternative gasoline after further testing or it could be blended with gasoline due to its high aromaticity and low oxygen content. In the end, using these wastes to produce valuable energetic oil is one step forward in the long journey towards ensuring energy security away from petroleum sources, in addition to the major contribution to waste management and landfill reduction.

## Figures and Tables

**Table 1:** Characterization of the parent, Fe-, Fe/Ni-, Ni-modified ZSM-5 zeolites.

	ZSM-5	Fe-ZSM-5	Fe/Ni-ZSM-5	Ni-ZSM-5
SiO_2_/Al_2_O_3_	38.0	38.4	38.9	38.5
Fe (wt.%) ^a^	0.04	1.46	1.32	0.04
Ni (wt.%) ^a^	-	-	1.22	1.21
BET surface area (m^2^/g) ^b^	282.0	299.1	284.9	274.0
Micropore surface area (m^2^/g) ^c^	109.5	135.5	123.8	126.8
External surface area (m^2^/g) ^c^	172.5	163.6	161.1	147.3
Specific pore volume (cm^3^/g) ^c^	0.24	0.24	0.23	0.22
Micropore volume (cm^3^/g) ^c^	0.05	0.06	0.06	0.06

^a^ Actual Fe and Ni loading measured by ICP-OES and XRF. ^b^ From N_2_ adsorption/desorption (BET). ^c^ From N_2_ adsorption/desorption (tplot).

**Table 2:** Chemical families of pyrolytic oil with their corresponding reference compound.

RT (min) *	Chemical Family	Reference Compound
6.63	Furans	Furan
11.15	Carboxylic acids	Acetic acid
12.13	Aromatics	Toluene
18.39	Esters & Ethers	Allyl butyrate
19.52	Aldehydes	Furfural
21.63	Ketones	2-methyl-2-cyclopenten-1-one
31.82	Phenols	p-cresol
40.76	PAH	Bibenzyl
41.74	Guaiacols	4-methylcatechol
42.74	Nitrogenates	Benzamide
49.58	Carbohydrates	Levoglucosan

* GC-FID retention time.

**Table 3:** Catalyst effect on the chemical family distribution and properties on BW bio-oil.

	Concentrations (wt.%)				
	No Catalyst	ZSM-5	Fe-ZSM-5	Fe/Ni-ZSM-5	Ni-ZSM-5
Carboxylic acids	35.3	35.7	33.5	36.1	35.3
Ester	5.8	4.5	4.4	4.7	4.4
Ether	0.7	1.0	0.9	0.9	0.9
Nitrogenates	4.4	3.5	3.8	4.3	4.1
Ketones	15.0	14.0	14.0	13.8	14.7
Furans	9.8	8.8	8.5	8.8	9.6
Aldehydes	2.4	0.5	0.5	0.7	0.8
Carbohydrates	17.3	9.5	11.7	12.5	12.4
Phenols	2.8	7.7	8.0	5.0	3.9
Guaiacols	5.2	6.3	7.0	6.6	6.9
Aromatics	-	2.8	2.6	2.6	2.7
PAH	-	3.9	3.3	2.3	2.1
Oxygen content	41.3	36.4	37.3	39.0	38.9
LHV (MJ/kg)	18.4	20.4	20.3	19.4	19.4
Liquid yield	65.0	56.2	56.0	47.9	56.3
Char yield	20.0	21.3	20.7	21.3	20.0
Coke	-	3.0	3.3	3.3	2.3
Gaz yield	15.0	19.5	20.0	27.5	21.4

**Table 4:** Catalyst effect on the chemical family distribution and properties on PS pyrolytic oil.

	Concentrations (wt.%)				
	No catalyst	ZSM-5	Fe-ZSM-5	Fe/Ni-ZSM-5	Ni-ZSM-5
Aromatics	76.7	82.2	77.7	81.7	77.8
PAH	23.3	17.8	22.3	18.3	22.2
LHV (MJ/kg)	40.6	40.6	40.0	40.6	40.5
Liquid yield	99.9	96.2	96.1	96.6	96.3
Char yield	0.0	0.0	0.0	0.0	0.0
Coke	0.0	3.3	3.1	3.0	3.3
Gaz yield	0.1	0.5	0.8	0.4	0.4

**Table 5:** Comparison with pyrolytic oils and between conventional fuels.

Name	LHV (MJ/kg)	LHV Change (%)	O% (wt.%)	O% Change (%)	Ref.
BW (No cat.)	18.4	n.a	41.3	n.a	This work
BW (ZSM-5)	20.4	+10.9 ^a^	36.4	−11.9 ^a^	
BW-PS 50-50 (No cat.)	36.5	+98.4	7.8	−81.1	
BW-PS 50-50 (Fe/Ni-ZSM-5)	38.8	+110.9	3.2	−92.3	
BW-PS 75-25 (No cat.)	30.0	+63.0	19.6	−52.5	
BW-PS 75-25 (Fe/Ni-ZSM-5)	35.8	+94.6	8.8	−78.7	
PS	40.6	n.a	-	n.a	
Gasoline	43.4–46.5		~2.7		[40,41,42]
Kerosene	43.0–46.2		n.a.		
Diesel	42.8–45.8		~1.8		

^a^ Catalyst and/or synergetic improvement on the LHV/O% relative to BW bio-oil.

**Table 6:** Evolution of BW-PS 50-50 (Fe/Ni-ZSM-5) liquid oil composition and properties relative to temperature (wt.%).

Chemical Families	Operating Temperature (°C) ± 5 °C			
	450	500	550	600
Carboxylic acids	3.6	2.9	4.1	5.6
Ketones	1.3	0.9	1.5	2.1
Furans	0.7	0.8	1.2	1.5
Carbohydrates	1.3	1.7	2.7	3.0
Aromatics	68.9	66.2	68.4	74.0
PAH	22.5	25.8	20.3	12.2
Gas yield	14.5	16.1	17.9	23.0
Liquid yield	70.2	69.9	66.1	61.0
Char yield	12.7	10.3	10.0	9.0
Coke	2.7	3.6	6.0	7.0
O%	3.6	3.2	4.7	6.0
LHV *	38.7	38.8	38.0	37.5

* Lower heating value (MJ/kg).

**Table 7:** Ultimate and proximate analysis of used raw materials (wt.%).

Name	Ultimate Analysis			Proximate Analysis			
	% C	% H	% O *	Moisture	Fixed Carbon	Volatile	Ash
PS	92.6	7.4	-	-	0.3	99.7	-
BW	47.4	6.1	46.5	5.7	17.5	75.9	0.9

* Oxygen percentage was calculated by difference.

**Table 8:** Summary of the performed experiments.

Reaction	Catalyst	BW-PS Percentage (wt.%)	Temperature (°C)
Biomass pyrolysis	No catalyst		500
	ZSM-5		
	Fe-ZSM-5	100-0	
	Fe/Ni-ZSM-5		
	Ni-ZSM-5		
Plastic pyrolysis	No catalyst		500
	ZSM-5		
	Fe-ZSM-5	0-100	
	Fe/Ni-ZSM-5		
	Ni-ZSM-5		
Co-pyrolysis	No catalyst	25-50-75	500
	No catalyst	50	450-500-550-600
	ZSM-5	50	500
	Fe-ZSM-5	50	
	Fe/Ni-ZSM-5	25-50-75	
	Ni-ZSM-5	50	
	Fe/Ni-ZSM-5	50	450-500-550-600

## Data Availability

Not applicable.

## Supplementary Materials

The following supporting information can be downloaded at: https://www.mdpi.com/article/10.3390/molecules28155758/s1, Table S1: List of the major products present in BW-PS catalytic pyrolytic oil.

## Abbreviations

**Abbreviations**	**Definition**	
FID	Flame-ionization detector	
TCD	Thermal conductivity detector	
GC-MS	Gas chromatography–mass spectroscopy	
PS PAH	Polystyrene Polycyclic aromatic hydrocarbon	
ECN	Effective carbon number	
TGA	Thermogravimetric analysis	
n.a.	Not applicable	
**Symbols**	**Symbol name**	**Unit**
m	Mass	[g]
z	Charge number	
(m/z)	Mass-to-charge ratio	[g·mol^−1^]
wt.%	Weight percentage	[%]
LHV	Lower heating value	[MJ·kg^−1^]
HHV	Higher heating value	[MJ·kg^−1^]
r^2^	Pearson’s coefficient of determination	
I.D./O.D.	Inner and outer diameter	[m]
a.u	Arbitrary unit	

## References

[1] Zhang H., Cheng F., Li Y., He C., Li H., Yang S. (2022). Polymeric organophosphate-hafnium unconventional MOFs nanohybrids enable high-efficiency upgrading of biomass feedstocks via cascade catalytic transfer hydrogenation-dehydration. Ind. Crops Prod..

[2] Li Y., Zhang S., Li Z., Zhang H., Li H., Yang S. (2022). Green synthesis of heterogeneous polymeric bio-based acid decorated with hydrophobic regulator for efficient catalytic production of biodiesel at low temperatures. Fuel.

[3] Abnisa F. (2013). Co-pyrolysis of palm shell and polystyrene waste mixtures to synthesis liquid fuel. Fuel.

[4] Czernik S., Bridgwater A.V. (2004). Overview of Applications of Biomass Fast Pyrolysis Oil. Energy Fuels.

[5] Zhang X. (2016). Catalytic co-pyrolysis of lignocellulosic biomass with polymers: A critical review. Green Chem..

[6] Roy P., Jahromi H., Rahman T., Baltrusaitis J., Hassan E.B., Torbert A., Adhikari S. (2023). Hydrotreatment of pyrolysis bio-oil with non-edible carinata oil and poultry fat for producing transportation fuels. Fuel Process. Technol..

[7] Yan W., Acharjee T.C., Coronella C.J., Vásquez V.R. (2009). Thermal pretreatment of lignocellulosic biomass. Environ. Prog. Sustain. Energy.

[8] Qi Z. (2007). Review of biomass pyrolysis oil properties and upgrading research. Energy Convers. Manag..

[9] Abnisa F., Wan Daud W.M.A. (2014). A review on co-pyrolysis of biomass: An optional technique to obtain a high-grade pyrolysis oil. Energy Convers. Manag..

[10] Bridgwater A.V. (2012). Review of fast pyrolysis of biomass and product upgrading. Biomass Bioenergy.

[11] Liu C., Wang H., Karim A.M., Sun J., Wang Y. (2014). Catalytic fast pyrolysis of lignocellulosic biomass. Chem. Soc. Rev..

[12] Ruddy D.A., Schaidle J.A., Iii J.R.F., Wang J., Moens L., Hensley J.E. (2014). Recent advances in heterogeneous catalysts for bio-oil upgrading via “ex situ catalytic fast pyrolysis”: Catalyst development through the study of model compounds. Green Chem..

[13] Iliopoulou E.F., Stefanidis S., Kalogiannis K., Psarras A.C., Delimitis A., Triantafyllidis K.S., Lappas A.A. (2014). Pilot-scale validation of Co-ZSM-5 catalyst performance in the catalytic upgrading of biomass pyrolysis vapours. Green Chem..

[14] Carlson T.R., Cheng Y.-T., Jae J., Huber G.W. (2011). Production of green aromatics and olefins by catalytic fast pyrolysis of wood sawdust. Energy Environ. Sci..

[15] Zhang H., Carlson T.R., Xiao R., Huber G.W. (2012). Catalytic fast pyrolysis of wood and alcohol mixtures in a fluidized bed reactor. Green Chem..

[16] Mohabeer C., Reyes L., Abdelouahed L., Marcotte S., Taouk B. (2019). Investigating catalytic de-oxygenation of cellulose, xylan and lignin bio-oils using HZSM-5 and Fe-HZSM-5. J. Anal. Appl. Pyrolysis.

[17] Zhang H., Nie J., Xiao R., Jin B., Dong C., Xiao G. (2014). Catalytic Co-pyrolysis of Biomass and Different Plastics (Polyethylene, Polypropylene, and Polystyrene) To Improve Hydrocarbon Yield in a Fluidized-Bed Reactor. Energy Fuels.

[18] Wang K., Kim K.H., Brown R.C. (2014). Catalytic pyrolysis of individual components of lignocellulosic biomass. Green Chem..

[19] Choi S.J., Park S.H., Jeon J.-K., Lee I.G., Ryu C., Suh D.J., Park Y.-K. (2013). Catalytic conversion of particle board over microporous catalysts. Renew. Energy.

[20] Wang Z., Burra K.G., Lei T., Gupta A.K. (2021). Co-pyrolysis of waste plastic and solid biomass for synergistic production of biofuels and chemicals-A review. Prog. Energy Combust. Sci..

[21] Zhang B., Zhong Z., Ding K., Song Z. (2015). Production of aromatic hydrocarbons from catalytic co-pyrolysis of biomass and high density polyethylene: Analytical Py–GC/MS study. Fuel.

[22] Xue Y., Kelkar A., Bai X. (2016). Catalytic co-pyrolysis of biomass and polyethylene in a tandem micropyrolyzer. Fuel.

[23] Vo T.A., Tran Q.K., Ly H.V., Kwon B., Hwang H.T., Kim J., Kim S.-S. (2022). Co-pyrolysis of lignocellulosic biomass and plastics: A comprehensive study on pyrolysis kinetics and characteristics. J. Anal. Appl. Pyrolysis.

[24] Engamba Esso S.B., Xiong Z., Chaiwat W., Kamara M.F., Longfei X., Xu J., Ebako J., Jiang L., Su S., Hu S. (2022). Review on synergistic effects during co-pyrolysis of biomass and plastic waste: Significance of operating conditions and interaction mechanism. Biomass Bioenergy.

[25] Anuar Sharuddin S.D., Abnisa F., Wan Daud W.M.A., Aroua M.K. (2016). A review on pyrolysis of plastic wastes. Energy Convers. Manag..

[26] Artetxe M., Lopez G., Amutio M., Barbarias I., Arregi A., Aguado R., Bilbao J., Olazar M. (2015). Styrene recovery from polystyrene by flash pyrolysis in a conical spouted bed reactor. Waste Manag..

[27] Jaafar Y., Abdelouahed L., Hage R.E., Samrani A.E., Taouk B. (2022). Pyrolysis of common plastics and their mixtures to produce valuable petroleum-like products. Polym. Degrad. Stab..

[28] Mullen C.A., Boateng A.A. (2015). Production of Aromatic Hydrocarbons via Catalytic Pyrolysis of Biomass over Fe-Modified HZSM-5 Zeolites. ACS Sustain. Chem. Eng..

[29] Sun L., Zhang X., Chen L., Zhao B., Yang S., Xie X. (2016). Comparision of catalytic fast pyrolysis of biomass to aromatic hydrocarbons over ZSM-5 and Fe/ZSM-5 catalysts. J. Anal. Appl. Pyrolysis.

[30] Matovic M.D. (2013). Biomass Now: Cultivation and Utilization.

[31] Yao W., Li J., Feng Y., Wang W., Zhang X., Chen Q., Komarneni S., Wang Y. (2015). Thermally stable phosphorus and nickel modified ZSM-5 zeolites for catalytic co-pyrolysis of biomass and plastics. RSC Adv..

[32] Lin X., Zhang Z., Sun J., Guo W., Wang Q. (2015). Effects of phosphorus-modified HZSM-5 on distribution of hydrocarbon compounds from wood–plastic composite pyrolysis using Py-GC/MS. J. Anal. Appl. Pyrolysis.

[33] Li J., Yu Y., Li X., Wang W., Yu G., Deng S., Huang J., Wang B., Wang Y. (2015). Maximizing carbon efficiency of petrochemical production from catalytic co-pyrolysis of biomass and plastics using gallium-containing MFI zeolites. Appl. Catal. B Environ..

[34] Li X., Li J., Zhou G., Feng Y., Wang Y., Yu G., Deng S., Huang J., Wang B. (2014). Enhancing the production of renewable petrochemicals by co-feeding of biomass with plastics in catalytic fast pyrolysis with ZSM-5 zeolites. Appl. Catal. A Gen..

[35] Dorado C., Mullen C.A., Boateng A.A. (2014). H-ZSM5 Catalyzed Co-Pyrolysis of Biomass and Plastics. ACS Sustain. Chem. Eng..

[36] Hassan E.B., Elsayed I., Eseyin A. (2016). Production high yields of aromatic hydrocarbons through catalytic fast pyrolysis of torrefied wood and polystyrene. Fuel.

[37] Mohabeer C., Reyes L., Abdelouahed L., Marcotte S., Buvat J.-C., Tidahy L., Abi-Aad E., Taouk B. (2019). Production of liquid bio-fuel from catalytic de-oxygenation: Pyrolysis of beech wood and flax shives. J. Fuel Chem. Technol..

[38] Valle B., Gayubo A.G., Aguayo A.T., Olazar M., Bilbao J. (2010). Selective Production of Aromatics by Crude Bio-oil Valorization with a Nickel-Modified HZSM-5 Zeolite Catalyst. Energy Fuels.

[39] Ding Y.-L., Wang H.-Q., Xiang M., Yu P., Li R.-Q., Ke Q.-P. (2020). The Effect of Ni-ZSM-5 Catalysts on Catalytic Pyrolysis and Hydro-Pyrolysis of Biomass. Front. Chem..

[40] Klein A., Bockhorn O., Mayer K., Grabner M. (2016). Central European wood species: Characterization using old knowledge. J. Wood Sci..

[41] Li X., Dong W., Zhang J., Shao S., Cai Y. (2020). Preparation of bio-oil derived from catalytic upgrading of biomass vacuum pyrolysis vapor over metal-loaded HZSM-5 zeolites. J. Energy Inst..

[42] Thommes M., Kaneko K., Neimark A.V., Olivier J.P., Rodriguez-Reinoso F., Rouquerol J., Sing K.S.W. (2015). Physisorption of gases, with special reference to the evaluation of surface area and pore size distribution (IUPAC Technical Report). Pure Appl. Chem..

[43] Botas J.A., Serrano D.P., García A., Ramos R. (2014). Catalytic conversion of rapeseed oil for the production of raw chemicals, fuels and carbon nanotubes over Ni-modified nanocrystalline and hierarchical ZSM-5. Appl. Catal. B Environ..

[44] Zhu L., Lv X., Tong S., Zhang T., Song Y., Wang Y., Hao Z., Huang C., Xia D. (2019). Modification of zeolite by metal and adsorption desulfurization of organic sulfide in natural gas. J. Nat. Gas Sci. Eng..

[45] Topaloǧlu Yazıcı D., Bilgiç C. (2010). Determining the surface acidic properties of solid catalysts by amine titration using Hammett indicators and FTIR-pyridine adsorption methods: Determining the surface acidic properties of solid catalysts. Surf. Interface Anal..

[46] Srivastava R., Srinivas D., Ratnasamy P. (2006). Sites for CO2 activation over amine-functionalized mesoporous Ti(Al)-SBA-15 catalysts. Microporous Mesoporous Mater..

[47] Ward J.W. (1970). Thermal decomposition of ammonium Y zeolite. J. Catal..

[48] Ferretto L., Glisenti A. (2003). Surface Acidity and Basicity of a Rutile Powder. Chem. Mater..

[49] Mohabeer C., Abdelouahed L., Marcotte S., Taouk B. (2017). Comparative analysis of pyrolytic liquid products of beech wood, flax shives and woody biomass components. J. Anal. Appl. Pyrolysis.

[50] Gucho E.M., Shahzad K., Bramer E.A., Akhtar N.A., Brem G. (2015). Experimental Study on Dry Torrefaction of Beech Wood and Miscanthus. Energies.

[51] Zhao C., Jiang E., Chen A. (2017). Volatile production from pyrolysis of cellulose, hemicellulose and lignin. J. Energy Inst..

[52] Mihalcik D.J., Mullen C.A., Boateng A.A. (2011). Screening acidic zeolites for catalytic fast pyrolysis of biomass and its components. J. Anal. Appl. Pyrolysis.

[53] Cheng Y.-T., Huber G.W. (2012). Production of targeted aromatics by using Diels–Alder classes of reactions with furans and olefins over ZSM-5. Green Chem..

[54] Dorado C., Mullen C.A., Boateng A.A. (2015). Origin of carbon in aromatic and olefin products derived from HZSM-5 catalyzed co-pyrolysis of cellulose and plastics via isotopic labeling. Appl. Catal. B Environ..

[55] Dyer A.C., Nahil M.A., Williams P.T. (2021). Catalytic co-pyrolysis of biomass and waste plastics as a route to upgraded bio-oil. J. Energy Inst..

[56] Aitani A.M., Cleveland C.J. (2004). Oil Refining and Products. Encyclopedia of Energy.

[57] Kamrin M.A., Wexler P. (2014). Gasoline. Encyclopedia of Toxicology.

[58] Scanlon J.T., Willis D.E. (1985). Calculation of Flame Ionization Detector Relative Response Factors Using the Effective Carbon Number Concept. J. Chromatogr. Sci..

[59] Marlair G., Cwiklinski C., Tewarson A. (1999). An analysis of some practical methods for estimating heats of combustion in fire safety studies. Interflam 99.

