# Supplementary material for: Upgrading Pyrolytic Oil via Catalytic Co-Pyrolysis of Beechwood and Polystyrene
Source: Molecules. 2023 Jul 30;28(15):5758. doi: 10.3390/molecules28155758 (PMC10420871; doi:10.3390/molecules28155758)
Supplement: Supplementary file 1 [file molecules-28-05758-s001.zip › molecules-2468336-supplementary.pdf]

S. Supplementary Materials

This part shows the different tables that are related to the different results discussed in the text but could not be included.

Table S1. List of the major products present in BW-PS catalytic pyrolytic oil.

Compounds	BW	BW-PS	PS
Furan	✓	✓	
1,2,3-Propanetriol, monoacetate	✓	✓	
Benzene	✓	✓	✓
2,5-dimethyl-Furan	✓	✓	
Acetic acid	✓	✓	
2-propanone, 1-hydroxy-	✓	✓	
Toluene	✓	✓	✓
2-Butene, 1-butoxy-, (E)-	✓	✓	
1,2-Ethanediol, monoacetate	✓		
Ethylbenzene		✓	✓
o-Xylene	✓	✓	✓
p-Xylene		✓	✓
Styrene	✓	✓	✓
Furfural	✓	✓	
2-Cyclopenten-1-one	✓		
Benzene, 2-propenyl-		✓	
α-Methylstyrene		✓	✓
2-Cyclopenten-1-one, 2-hydroxy-	✓	✓	
Indane		✓	✓
Benzene, (1-methylenepropyl)-	✓	✓	
Indene	✓	✓	✓
Phenol	✓	✓	
Guaiacol	✓	✓	
Naphthalene	✓	✓	✓
Orthocresol	✓	✓	
3-methylphenol	✓	✓	
Phenol, 2,6-dimethyl-	✓	✓	
Naphthalene, 2-methyl-	✓	✓	✓
Naphthalene, 1-methyl-	✓	✓	✓
Eugenol	✓	✓	
Levoglucosan	✓	✓	
